# Nationwide Epic Cosmos Data Reveals Antidepressant Prescription Rate Trends Across Demographic Groups

**DOI:** 10.21203/rs.3.rs-9680849/v1

**Published:** 2026-05-28

**Authors:** Dmitry Scherbakov, Brandy Davis, Jeffrey Korte, Jihad S. Obeid, Alexander V. Alekseyenko

**Affiliations:** Medical University of South Carolina; Auburn University; Medical University of South Carolina; Medical University of South Carolina; Medical University of South Carolina

**Keywords:** Antidepressants, Depression, Epic Cosmos, Electronic health records, Sex differences, Life course

## Abstract

**Purpose:**

Sex disparities in depression and its treatment are well-documented, but less is known about how these patterns vary across the lifespan and intersect with race, neighborhood vulnerability, and prior health challenges.

**Methods:**

We analyzed data from 2.5 million patients sampled from the Epic Cosmos Electronic Health Record (EHR) database. Active antidepressant prescriptions were used as a proxy for depression diagnoses. Data were stratified by age, sex, race/ethnicity, residence, Social Vulnerability Index (SVI 2018), prior antidepressant use, and high-risk behaviors (HIV and chlamydia testing). Rates of prescriptions by sex and odds ratios (OR) for females versus males across age at time of visit were calculated.

**Results:**

The probability of receiving an antidepressant prescription rose sharply during adolescence, but more steeply for females, with female vs. male differences peaking at ages 17–18 (OR = 2). In the overall population, the sex gap narrowed in young adulthood (OR = 1.6 at ages 25–30), remained stable through mid-life, and widened again after age 55 (OR = 1.75 by age 70). However, the later-life increase was not observed uniformly across some groups, such as the unmarried, divorced, and living in vulnerable areas. The white population had noticeably higher prescription rates across the life span, while the Asian group had the highest adolescent peak (female vs. male OR = 2.75). Prior HIV and chlamydia testing strata demonstrated higher antidepressant prescribing and a larger, earlier adolescent female-to-male OR, although this factor likely explains only a small portion of the overall adolescent sex difference at the population level.

**Conclusions:**

The well-known depression sex gap in adolescents observed in the general population, and the lesser-known retirement gap, are shaped by a combination of demographic, economic, and behavioral factors, explored in this study.

## BACKGROUND

Disparities in depression prevalence rates across sex, race, and ethnicity are well documented. For example, C. Kuehner [1] reviewed epidemiological studies and found that women are approximately twice as likely as men to experience depression, a pattern observed across cultures and age groups. Williams et al. [2] demonstrated that African American and Hispanic populations in the United States report higher rates of depressive symptoms but are less likely to receive adequate treatment compared to non-Hispanic White populations. Alegría et al. [3] further highlighted that socioeconomic status and neighborhood disadvantage significantly contribute to racial and ethnic disparities in depression prevalence and outcomes.

Less is known about how these social factors combine over time to shape sex differences in depression across the life span. Salk et al. [4] presented results of a meta-analysis that revealed patterns in disparities of depression outcomes between females and males, binning data into age groups, with subgroup analysis comparing high- and low-income countries. The study used representative national samples across different countries and was able to demonstrate that higher-income countries have a more pronounced sex gap in depression prevalence, with females exhibiting higher rates and severity than males, particularly during adolescence.

Prior treatment history and health-related behaviors may further complicate these disparities. Individuals with a previous antidepressant course have a markedly higher likelihood of receiving subsequent prescriptions, reflecting the chronic–recurrent nature of depression and guideline-based continuation strategies. Longitudinal studies show that a single depressive episode predicts at least a 50% risk of recurrence, with women more likely than men to experience chronic depression and receive long-term treatment [5]. Risk-related behaviors, such as sexual risk-taking, have also been linked to higher depressive symptoms and increased health care use, particularly among adolescents and young adults [6–8]. These lines of evidence suggest that prior health experiences can amplify existing sex differences in depression.

In this study, we pursued three main goals. First, we address a future research direction proposed by Salk et al. [4], namely, to identify the individual trajectories of the two sexes (female and male) across the lifespan using the Epic Cosmos (Epic Systems, Verona, WI) electronic health record (EHR) database, which contains data on over 300 million patients (> 99% receiving care in the US), using antidepressant prescription as a proxy for depression diagnosis [9]. Second, we sought to validate the shape of the female-to-male odds ratio curve – characterized by a peak in adolescence as reported by Salk et al. and replicated in our automated meta-analysis [10], which identified an additional peak close to retirement age. Third, we examined sex differences in antidepressant prescribing at the intersection of race/ethnicity, neighborhood social vulnerability, marital status, and selected health history indicators (prior antidepressant use and recent HIV or chlamydia testing). By integrating these factors, our study aims to provide a more nuanced understanding of how depression trajectories and sex disparities differ across both social and health-related dimensions.

## METHODS

The Epic Cosmos EHR database was used to query over 1.8 million patients of different ages, alongside their visit history up to 7 years before the date of extraction (June 2025) and history of antidepressant prescriptions (defined as Epic Grouper “Antidepressant medications” covering over 700 medications), 12 months before and 12 months after each visit.

The dataset for the analysis used patient-visit information, and age at visit was calculated, and only visits with distinct floored ages per patient were included (thus, multiple visits of each patient were allowed if the patient’s age floored was different at each visit). In addition, the number of visits 12 months before and after each visit was calculated, as well as antidepressant prescriptions, and HIV and chlamydia testing encounters (used as proxies for risky health behaviors [11]). The following CPT codes for HIV and chlamydia testing were used: 86631, 86632, 87810, 3511F, 87110. 87320, 87270, 87486,87485, 87487, 87491, 87490, 87492; 0575F, 3502F, 3503F, 3292F, S3645, 87390, 87389, 87391, 87806, 87535, 87534, 87536, 87538, 87537, 87539, 87906, 87901, 87904, 87903, 87903, 87904, G0435, 86701, 86703, 86702, 86689, G0432, G0433, G0475, and 3491F).

The following demographics were also extracted at the patient level and propagated to each visit: First Race (representing one of several possible race/ethnicity choices provided by the patient), Age (current and at visit), Sex at birth, and Marital status. Marital status in Epic Cosmos is provided only as the latest value, but since half of the visits occurred within 3 years before the extraction date, the marital status was still included in the analysis, keeping in mind the value may not match marital status at the visit, especially for visits at a younger age (under age 20), where individuals are mostly single.

The primary variable of interest was the proportion of visits after which individuals received antidepressant prescriptions (within 12 months). To calculate this proportion, the resulting dataset was analyzed by grouping by floored age (at visit) and sex, and stratifying by different variables to calculate and plot the proportion of individuals at each age (at visit) year who received a prescription, with an additional overlaying odds ratio plot comparing females vs males odds to receive an antidepressant prescription in the next 12 months. Spline smoothing was used alongside data points to enhance the readability of the figures. The confidence interval was calculated using the Wald method and is displayed using ribbons.

For analysis of HIV and chlamydia testing we enforced additional criteria on the sample: requirement to have at least one more visit in 12 months prior to anchor visit. This was done to balance possible healthcare utilization and data missingness differences (e.g. if patient had a testing procedure in 12 months prior to anchor, that means for untested patients we want to be sure we at least have any visit information in the same period)

## RESULTS

A total of 1,872,463 patients (53.2% female, Age at visit M = 45.7 years, SD = 22.0) contributed information on 6,445,919 visits.

### Overall life-course pattern

**Figure 1:**
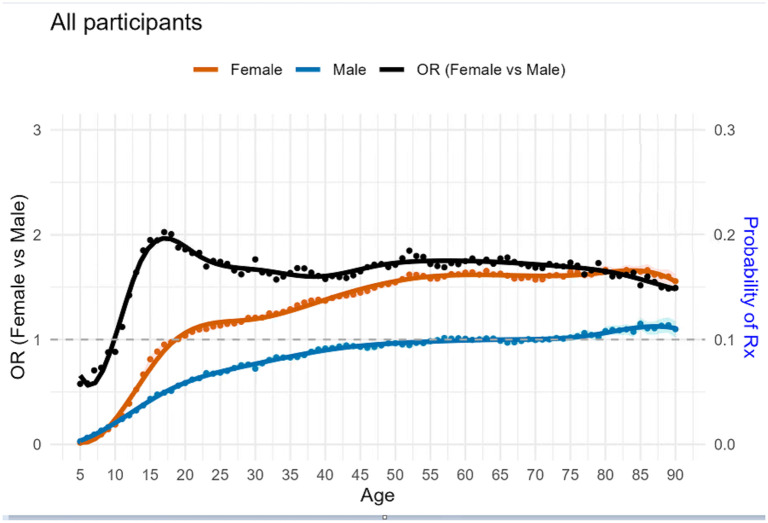
General population by Probability and OR (Female vs Male) to receive antidepressant prescription depending on age at visit. The thick line shows the spline approximation of individual data points; ribbons show the confidence intervals.

The probability of receiving an antidepressant prescription rose sharply for both sexes during adolescence, but more steeply for females. The peak female-to-male odds ratio (OR) was approximately 2 at floored age at visit 17–18 (Fig. 1) – indicating that adolescent females were nearly twice as likely to receive an antidepressant prescription. The gap narrowed in young adulthood, with the OR approaching 1.6 at ages 25–30, suggesting a reduced but still notable difference between sexes. However, after age 55, the gap widened again, with the OR increasing and reaching approximately 1.75 by age 70.

### By neighborhood SVI

**Figure 2:**
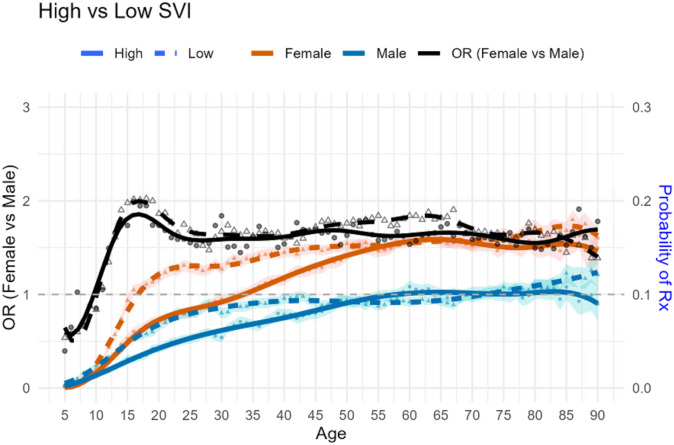
Patients stratified by sex and SVI of the residence, High (vulnerable) > 0.75, Low (non-vulnerable) < 0.25. Circles stand for individual OR curve points for the High SVI group, triangles for the Low SVI group.

Patients residing in the most vulnerable neighborhoods (SVI > 0.75) had the lowest absolute antidepressant prescribing rates for both females and males (Fig. 2, solid red and blue lines). In these high-SVI areas, the sex gap in prescribing was slightly attenuated, with the peak female-to-male odds ratio (solid line) reaching approximately 1.8 during adolescence, while the “retirement gap” was attenuated, with OR hovering at approximately 1.6. In contrast, patients in the least vulnerable neighborhoods (SVI < 0.25, dashed lines) experienced higher overall prescribing rates, and the sex gap was more pronounced, with a peak OR = 2 and a more pronounced retirement gap. These results suggest that both absolute prescribing rates and the magnitude of sex differences are influenced by neighborhood vulnerability.

### Race and ethnicity

White participants had noticeably higher prescription rates across the life span for both sexes, with an OR curve resembling that of the low SVI group (Fig. 2) with pronounced adolescent and retirement peaks. The Black/African American group didn’t show a retirement peak on the OR curve, but instead had an adulthood elevation (40–45 year olds) with a slight dip afterwards. For the Asian group, OR elevation occurred around age 50, followed by a retirement dip at 65. The Asian group also had a sharper adolescent peak (with individual OR reaching data points reaching 2.75), followed by a sharper decline and OR lower than that of the White in the adulthood/retirement period. Hispanic or Latino participants demonstrated adulthood elevation followed by slight fluctuations in the OR curve, without a significant retirement elevation or dip.

### Marital status

**Figure 4:**
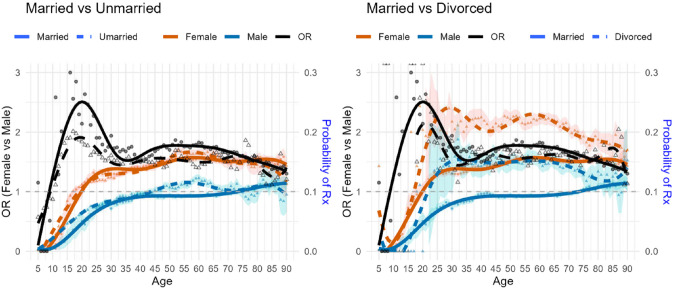
Participants were stratified by current marital status and age. Circles represent OR curve points for Married group. Note: Since marital status in the data only has the latest (current as of June 2025) update value, it can differ from the status at the time of visit that is used to calculate the probability of prescription by an average of three years.

Married (Fig. 4) individuals showed the most pronounced adolescent and retirement gaps. The former gap should be interpreted cautiously since marital status may be lagged by up to 7 years (3 years on average) in our study. The retirement gap was absent in the Unmarried group. Divorced participants had higher prescription rates than married and unmarried individuals, with the latter having comparable prescription rates to the married group.

### Prior antidepressant use

**Figure 5:**
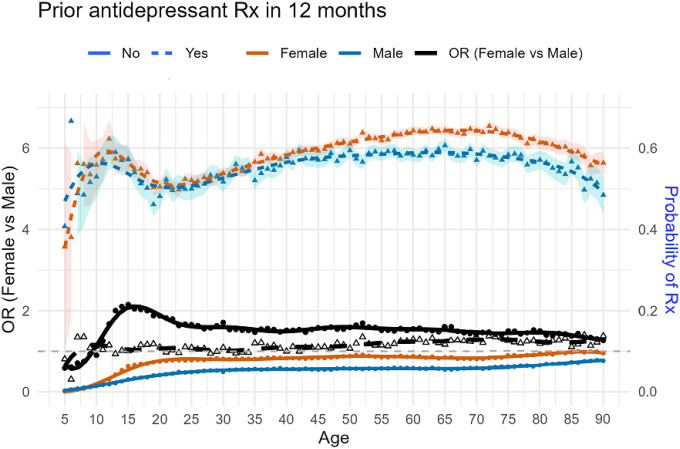
By prior (12 months) antidepressant prescription

Among individuals with a prior antidepressant prescription in the past year from the anchor visit (Fig. 5), the probability of receiving a new prescription rose sharply during adolescence for both sexes. This trend repeated during retirement age, where the increase was more pronounced for females. The overall sex difference was small for the prior prescription group, elevating to OR ≈ 1.25 in the retirement period. On the other hand, patients without prior antidepressant prescriptions showed lower prescription rates in the next 12 months, with the sex difference characterized by a strong adolescent peak (OR ≈ 2.2) and a notable absence of retirement elevation.

### STI Testing History: HIV and Chlamydia

**Figure 6:**
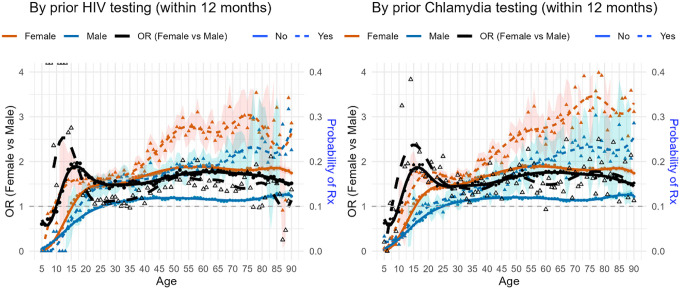
By prior HIV (left) and chlamydia (right) testing among patients with at least 1 visit within 12 months of the anchor visit. These figures shouldn’t be compared directly to previous ones, which don’t have prior visit count filter applied.

Among visits which were preceded by at least one additional visit within 12 months, 2.9% of all visits had HIV testing within 12 months, 5.7% for 16–29-year-olds; 3.0% of all visits had chlamydia testing within 12 months, 9.0% for 16–29-year-olds. Among individuals with HIV or chlamydia testing within 12 months prior to the anchor visit (Fig. 6), the probability of receiving an antidepressant prescription increased more rapidly during adolescence, followed by a slow growth and sharp rise after age 30, which continued into late life. The adolescent peak female-to-male OR was higher, reaching values > 2.5, and in the HIV tested group earlier (before 15) than in the overall population. The OR curve around retirement had different shape and timing in the tested groups compared to untested. The no testing group mirrored the general population trends for participants with the same number of visits (≥ 1 visits in prior 12 months, general population figure with this filter by number of prior visits not shown), with females showing a very small increase in general population prescriptions, and hence there was a small increase of approx. 0.1 in the OR curve for 14–25 year olds.

## DISCUSSION

This study used data from more than 2.5 million patients in the Epic Cosmos EHR database to examine antidepressant prescribing patterns across the life span, with a focus on differences by sex, race, and ethnicity, neighborhood vulnerability, and prior health behaviors. Our findings confirm well-known sex differences in depression treatment and extend previous research by showing how these differences vary across social and health contexts.

Consistent with prior epidemiological studies and meta-analyses [1, 4, 10], we observed that females were more likely than males to receive antidepressant prescriptions at nearly all ages. The sex gap was largest during adolescence, when females were nearly twice as likely as males to receive a prescription, narrowed during young adulthood, then widened again in later life. This pattern aligns with earlier research showing that adolescent and young adult females are at higher risk for depression, likely due to a mix of biological changes, social pressures, and cultural factors.

The size of the sex gap in our study was somewhat smaller than that reported by Salk et al. [4], who found bigger differences—especially in early adolescence. This difference is likely due to how depression was measured. While the mentioned meta-analysis focused on symptoms and diagnoses of major depression, our study used antidepressant prescriptions as a proxy for depression. This approach captures initial and recurrent pharmacological treatments but is likely to miss untreated or non-pharmacologically treated depression.

Findings related to retirement age highlight the complexity of depression later in life. Prior studies have reported mixed results, with some showing increased depressive symptoms but no increase in diagnoses, others showing a decrease after retirement, and still others reporting higher antidepressant use among older women [12, 13]. In contrast, we found that antidepressant prescribing increased for both sexes during retirement age, more steeply for females which results in the retirement gap for ORs observed in the overall and White population, with some variations of timing and magnitude in other demographic groups. This suggests that retirement-related changes—such as shifts in social roles, daily structure, financial stress, or health status—may affect men and women differently [14, 15].

Neighborhood-level vulnerability also played an important role. Patients living in the most socially vulnerable neighborhoods had lower overall rates of antidepressant prescribing for both sexes. In these areas, the sex gap was smaller than in less vulnerable neighborhoods. This pattern closely mirrors findings from Salk et al. [4], who observed smaller sex differences in countries with lower GDP. Together, these results suggest that limited access to mental health care in disadvantaged settings may reduce both overall treatment rates and observable sex differences, rather than indicating lower underlying need.

Racial and ethnic differences further supported this interpretation. White patients had higher prescription rates than other groups across the life span, consistent with prior research showing that racial and ethnic minority populations face greater barriers to mental health care [2, 3, 16]. While females consistently had higher prescribing rates than males across all racial/ethnic groups, the size and shape of the sex gap varied. Asian patients showed the most pronounced adolescent peak in the female-to-male odds ratio; also, Asian and Hispanic/Latino individuals showed an increase in the OR gap during earlier adulthood compared to White individuals, where the gap was observed at retirement. These differences likely reflect a combination of structural barriers, cultural attitudes toward mental health treatment, and differences in access to care.

Stratifying the analysis by prior (within 12 months of the anchor visit date) antidepressant use and by health behaviors provided additional insight. Among patients with prior antidepressant prescriptions, overall prescribing rates were significantly higher, and sex differences were small, except during retirement age, when they started to be visible. This pattern is consistent with prior findings showing smaller symptom severity differences among individuals with existing mental health conditions [10]. The absence of a retirement-related sex gap in patients without prior antidepressant use, combined with its presence among those with prior use, can be explained by treatment continuation patterns – such as men being more likely to discontinue medication earlier than women [17]. This could explain the retirement gap observed in the general population (Fig. 1).

Patients with HIV or chlamydia testing within 12 months before the anchor visit showed earlier and larger sex differences during adolescence. This should be interpreted cautiously, as STI testing is strongly influenced by screening guidelines and routine reproductive health care, particularly among adolescent females [19]. As a result, testing may function as a proxy for healthcare engagement. Increased contact may provide more opportunities for depression screening and antidepressant prescribing, which could partially explain the larger female-to-male odds ratios observed in this subgroup. The association between STI testing and higher antidepressant prescribing may reflect increased healthcare engagement, reproductive health visits, or screening-related contact with providers, rather than behavioral risk itself [18]. Future analysis should focus on more types of testing, distinguishing between high-risk testing (e.g., HIV, syphilis) and recommended screenings.

### Limitations

Several important limitations should be considered when interpreting these findings. First, antidepressant prescriptions were used as a proxy for depression diagnosis. While this approach captures treated cases and recurrent episodes, it misses depression treated non-pharmacologically, as well as conflates the data with non-depression conditions, which are treated by antidepressant medications. Second, the analysis relied on electronic health record (EHR) data from the Epic Cosmos database, which may be subject to documentation biases, incomplete capture of care outside participating systems, and potential misclassification of demographic variables. We used the FirstRace variable from Epic Cosmos to identify the race and ethnicity, which doesn’t completely represent the racial profile of a participant, due to the possibility of identifying with multiple races in Epic. Third, marital status was included as a covariate, but its accuracy may be limited for younger patients, because Epic only contains the latest update of this information for each patient, which may differ from marital status at the time of visit used for the analysis, and may be inaccurate in general. Finally, Epic does not include sexual orientation data, which limits interpretation of findings related to STI testing and sex differences.

### Conclusion

This large-scale study demonstrates that the sex differences in antidepressant prescribing peak during adolescence and retirement and vary by race, neighborhood vulnerability, and prior health behaviors, but persist across all groups. Future research should integrate prescription data with diagnosis data for depression and explore additional biological and social determinants of health to better understand and address sex disparities in mental health during adolescence, retirement, and across the life span.

## Figures and Tables

**Figure 3:**
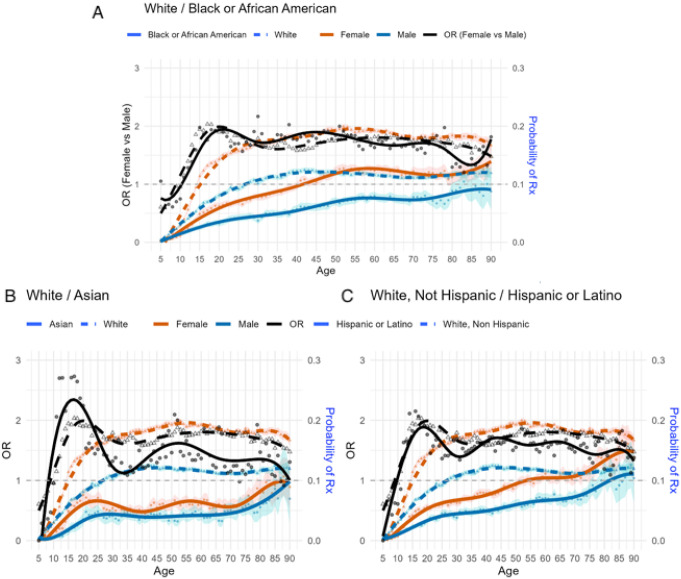
Participants stratified by race/ethnicity and sex. Circles represent the OR curve points for the White group.

## Data Availability

The datasets analyzed during the current study are not publicly available because they were obtained from the Epic Cosmos electronic health record platform under institutional access restrictions. Analytic code is stored in Epic cloud and can not be distributed, however corresponding author will provide analysis plan based on a reasonable request.

## References

[1] Kuehner C (2017) Why is depression more common among women than among men? lancet psychiatry 4(2):146–158 27856392 10.1016/S2215-0366(16)30263-2

[2] Williams DR, Gonzalez HM, Neighbors H, Nesse R, Abelson JM, Sweetman J, Jackson JS (2007) Prevalence and distribution of major depressive disorder in African Americans, Caribbean blacks, and non-Hispanic whites: results from the National Survey of American Life. Arch Gen Psychiatry 64(3):305–315 17339519 10.1001/archpsyc.64.3.305

[3] Alegría M, Chatterji P, Wells K, Cao Z, Chen C-n, Takeuchi D, Jackson J, Meng X-L (2008) Disparity in depression treatment among racial and ethnic minority populations in the United States. Psychiatric Serv 59(11):1264–1272 10.1176/appi.ps.59.11.1264 PMC2668139 18971402

[4] Salk RH, Hyde JS, Abramson LY (2017) Gender differences in depression in representative national samples: Meta-analyses of diagnoses and symptoms. Psychol Bull 143(8):783–822 28447828 10.1037/bul0000102 PMC5532074

[5] Solomon DA, Keller MB, Leon AC, Mueller TI, Lavori PW, Shea MT, Coryell W, Warshaw M, Turvey C, Maser JD (2000) Multiple recurrences of major depressive disorder. Am J Psychiatry 157(2):229–233 10671391 10.1176/appi.ajp.157.2.229

[6] Brown LK, Tolou-Shams M, Lescano C, Houck C, Zeidman J, Pugatch D, Lourie KJ, Group PSS (2006) Depressive symptoms as a predictor of sexual risk among African American adolescents and young adults. J Adolesc Health 39(3):444 e441–444. e448 10.1016/j.jadohealth.2006.01.015 16919811

[7] Hallfors DD, Iritani BJ, Miller WC, Bauer DJ (2007) Sexual and drug behavior patterns and HIV and STD racial disparities: the need for new directions. Am J Public Health 97(1):125–132 17138921 10.2105/AJPH.2005.075747 PMC1716241

[8] Shrier LA, Schillinger JA, Aneja P, Rice PA, Batteiger BE, Braslins PG, Orr DP, Fortenberry JD (2009) Depressive symptoms and sexual risk behavior in young, chlamydia-infected, heterosexual dyads. J Adolesc Health 45(1):63–69 19541251 10.1016/j.jadohealth.2008.11.016

[9] Mankowski MA, Bae S, Strauss AT, Lonze BE, Orandi BJ, Stewart D, Massie AB, McAdams-DeMarco MA, Oermann EK, Habal M (2025) Generalizability of kidney transplant data in electronic health records—The Epic Cosmos database vs the Scientific Registry of Transplant Recipients. Am J Transplant 25(4):744–755 39550008 10.1016/j.ajt.2024.11.008 PMC11972892

[10] Scherbakov D, Heider PM, Obeid JS, Alekseyenko AV, Lenert LA (2025) Gender Differences in Depression Symptom Severity: An AI-expedited Meta-analysis

[11] Veličko I, Ploner A, Sparén P, Marions L, Herrmann B, Kühlmann-Berenzon S (2016) Sexual and testing behaviour associated with Chlamydia trachomatis infection: a cohort study in an STI clinic in Sweden. BMJ open 6(8):e011312 10.1136/bmjopen-2016-011312 PMC5013445 27566631

[12] Gueltzow M, Bijlsma MJ, van Lenthe FJ, Myrskylä M (2023) The role of labor market inequalities in explaining the gender gap in depression risk among older US adults. Soc Sci Med 332:116100 37515952 10.1016/j.socscimed.2023.116100

[13] Hed S, Berg AI, Hansson I, Kivi M, Waern M (2024) Depressive symptoms across the retirement transition in men and women: associations with emotion regulation, adjustment difficulties and work centrality. BMC Geriatr 24(1):643 39085792 10.1186/s12877-024-05228-2 PMC11292945

[14] Dang L, Ananthasubramaniam A, Mezuk B Spotlight on the challenges of depression following retirement and opportunities for interventions. Clin Interv Aging 2022:1037–1056 35855744 10.2147/CIA.S336301 PMC9288177

[15] Zhai L, Wang J, Liu Y, Zhang H (2022) Involuntary retirement and depression among adults: A systematic review and meta-analysis of longitudinal studies. Front Psychiatry 13:747334 35185644 10.3389/fpsyt.2022.747334 PMC8854640

[16] Simpson SM, Krishnan LL, Kunik ME, Ruiz P (2007) Racial disparities in diagnosis and treatment of depression: a literature review. Psychiatr Q 78(1):3–14 17102936 10.1007/s11126-006-9022-y

[17] Pratt LA, Brody DJ, Gu Q (2017) Antidepressant Use among Persons Aged 12 and Over: United States, 2011–2014. NCHS Data Brief. Number 283. National Center for Health Statistics 29155679

[18] Vasilenko SA (2017) Age-varying associations between nonmarital sexual behavior and depressive symptoms across adolescence and young adulthood. Dev Psychol 53(2):366 27854469 10.1037/dev0000229 PMC5293671

[19] Force UPST, Davidson KW, Barry MJ, Mangione CM, Cabana M, Caughey AB, Davis EM, Donahue KE, Doubeni CA, Krist AH (2021) Screening for chlamydia and gonorrhea: US Preventive Services Task Force recommendation statement. JAMA 326(10):949–956 34519796 10.1001/jama.2021.14081

[20] Kong FY, Guy RJ, Hocking JS, Merritt T, Pirotta M, Heal C, Bergeri I, Donovan B, Hellard ME (2011) Australian general practitioner chlamydia testing rates among young people. Med J Aust 194(5):249–252 21381999 10.5694/j.1326-5377.2011.tb02957.x

